# Supplementary material for: Cold Atmospheric Plasma Jet Treatment Improves Human Keratinocyte Migration and Wound Closure Capacity without Causing Cellular Oxidative Stress
Source: Int J Mol Sci. 2022 Sep 13;23(18):10650. doi: 10.3390/ijms231810650 (PMC9504313; doi:10.3390/ijms231810650)
Supplement: Supplementary file 1 [file ijms-23-10650-s001.zip › ijms-1874551-supplementary.pdf]

Article

Cold Atmospheric Plasma Jet Treatment Improves Human Keratinocyte Migration and Wound Closure Capacity without Causing Cellular Oxidative Stress

Aurélie Marches, Emily Clement, Géraldine Albérola, Marie-Pierre Rols, Sarah Cousty, Michel Simon * and Nofel Merbahi

SUPPLEMENTARY MATERIALS

1. Supplementary Methods

Hydrogen peroxide (H_2O_2) quantification. The concentration of H_2O_2 in DPBS was quantified immediately after exposure to plasma using the Amplex® Red Hydrogen Peroxide/Peroxidase Assay Kit (Invitrogen, ThermoFisher Scientific) according to the manufacturer's protocols. Absorbance at 560 nm was read using a plate reader (Biochrom Asys UVM340, Biochrom Ltd, Cambridge, United Kingdom).

Nitrite/nitrate (NO_2^-/NO_3^-) ions quantification. NO_2^-/NO_3^- ion concentrations in DPBS were quantified immediately after exposure to the plasma jet using a colorimetric Nitrite/Nitrate Assay kit (Sigma, Merck KGaA, Darmstadt, Germany) according to the manufacturer's protocols. Absorbance at 540 nm was read using the Biochrom Asys UVM340 plate reader.

2. Supplementary Figures

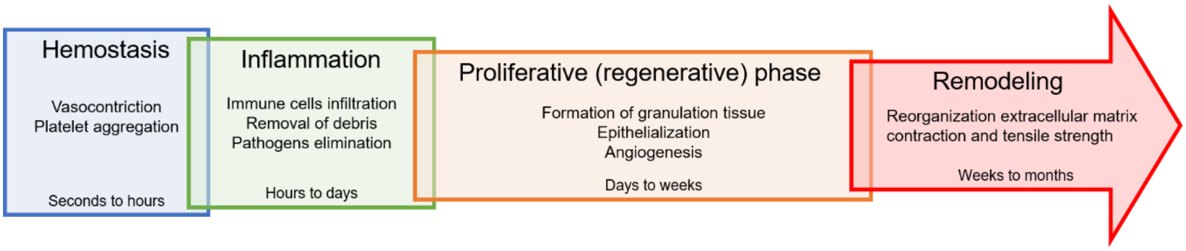

Figure S1: Diagram of the four stages of acute wound healing.

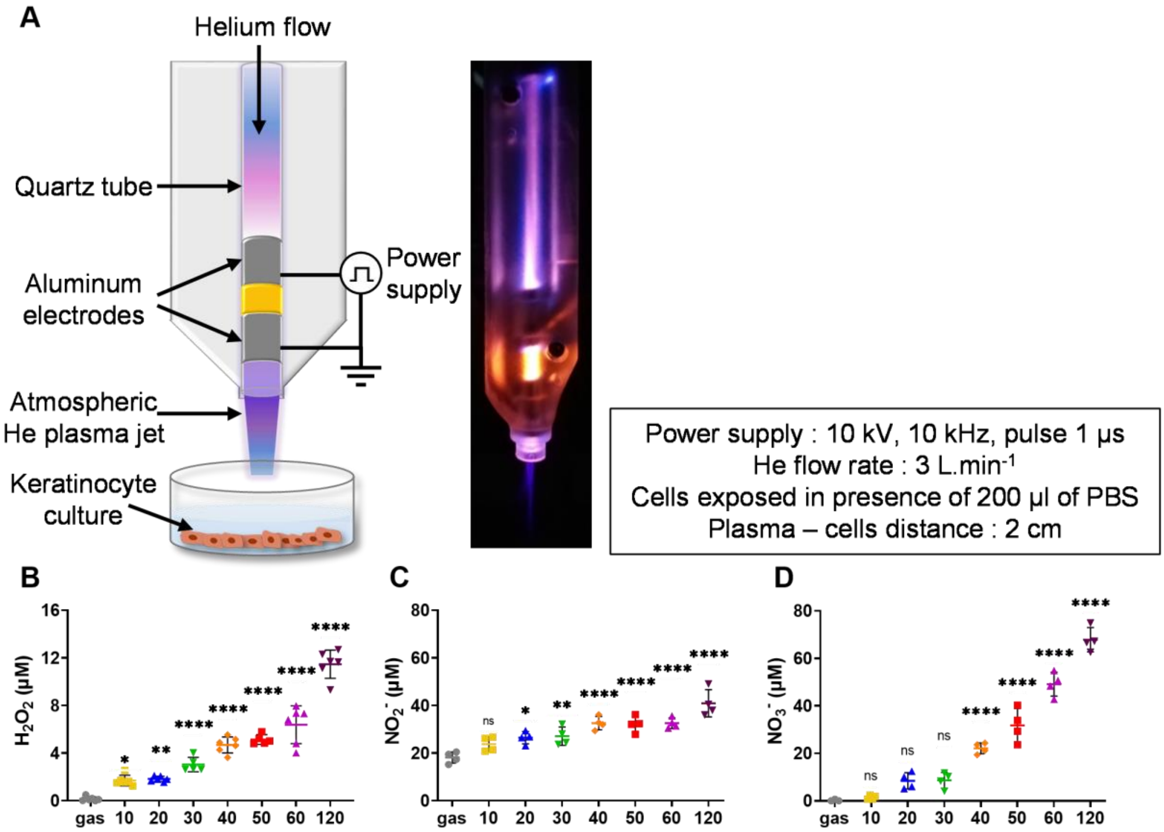

Figure S2: Concentrations of reactive oxygen and nitrogen oxide species increased with increasing exposure time to the helium plasma jet. A. Schematic diagram of the CAP-treatment using our helium plasma jet device. **B-D.** Dosages by colorimetric assays of RONS in fresh PBS, containing $CaCl_2$ and $NaCl_2$, CAP-treated for 10 s to 120 s, treated with gas alone for 120 s as control: **B.** Hydrogen peroxide, **C.** Nitrite anion, **D.** Nitrate anion. n > 4; error bars represent S.D. *, *$p < 0.05$*; **, *$p < 0.005$*; ***, *$p < 0.001$*; ****, *$p < 0.0001$*.

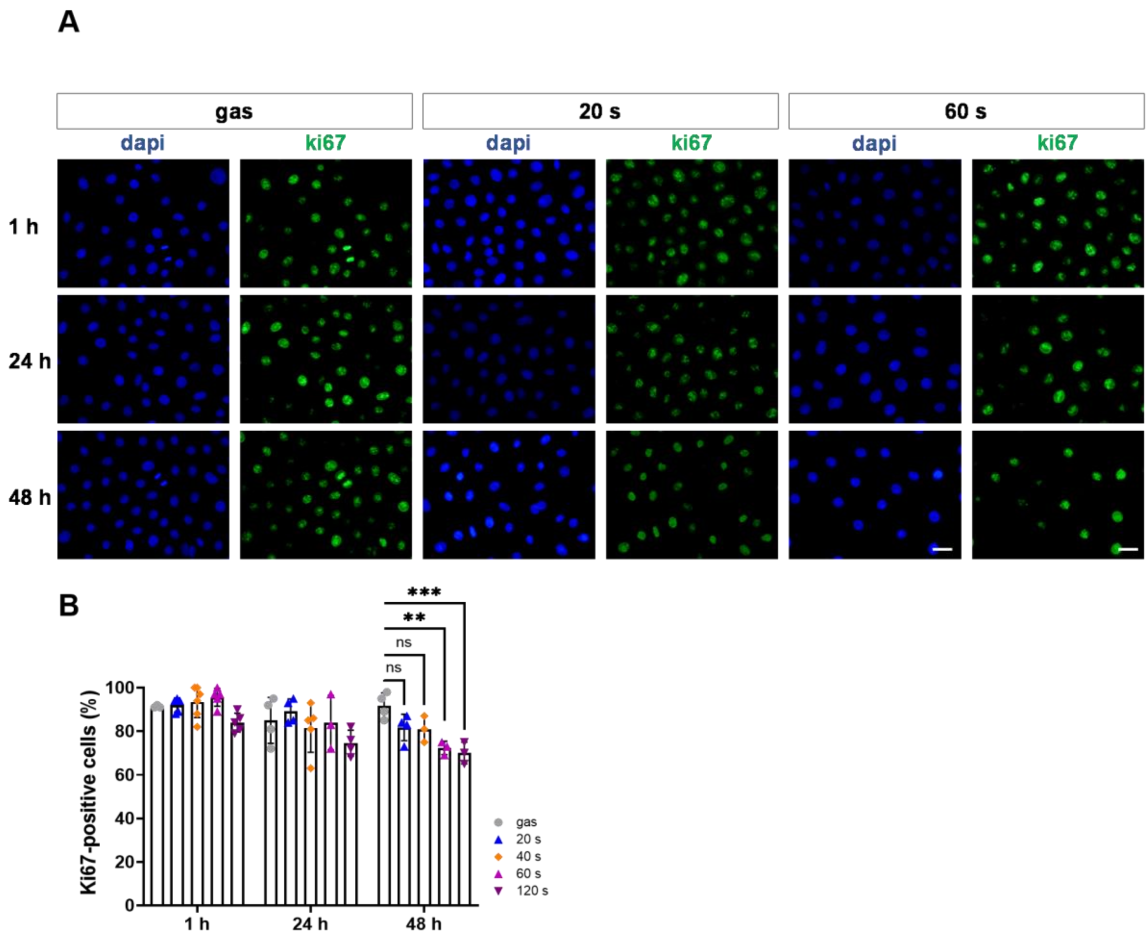

Figure S3: CAP treatments of less than 60 s did not affect N/TERT-1 proliferation. N/TERT-1 keratinocytes were CAP-treated for the indicated periods of time ranging from 20 s to 120 s. The proportion of proliferating cells was determined by immunocytochemistry on fixed and permeabilized cells using an anti-Ki-67 antibody (green), 1 h, 24 h and 48 h after exposure to CAP. Nuclei were stained with DAPI (blue). **A.** Representative images. White arrows indicate Ki-67 negative cells. Scale bar is 25 µm. **B.** Quantitative analyses. N = 4; error bars represent S.D. *, $p < 0.05$; **, $p < 0.005$; ***, $p < 0.001$; ****, $p < 0.0001$. ns: not significant.

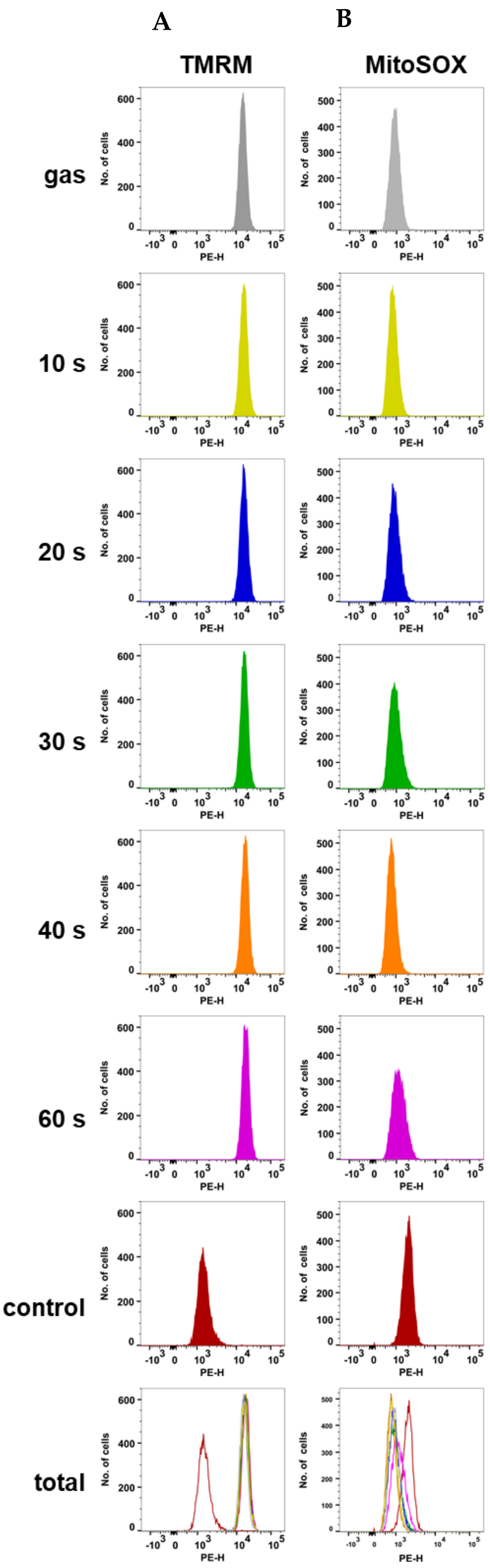

Figure S4: Representative graphs of flow cytometry quantification using TMRM and MitoSOX 6 h after exposure to CAP. N/TERT-1 keratinocytes were exposed to CAP for the indicated periods of time ranging from 10 s to 60 s. **A.** Representative graphs of flow cytometry quantification of TMRM immunofluorescence 6 h after exposure to CAP. We used 2 µM CCCP as a positive control for membrane depolarization. **B.** Representative graphs of flow cytometry quantification of MitoSOX Red Mitochondrial SuperOXide immunofluorescence 6 h after exposure to CAP. Two µM antimycin was used as a positive control for mitochondrial anion superoxide production.
